# Mitochondrial genome of *Diaphorencyrtus aligarhensis* (Hymenoptera: Chalcidoidea: Encyrtidae) and phylogenetic analysis

**DOI:** 10.1080/23802359.2019.1667913

**Published:** 2019-09-23

**Authors:** Yimin Du, Xiang Song, Xinjun Liu, Balian Zhong

**Affiliations:** a School of Life Sciences, Gannan Normal University, Ganzhou, Jiangxi, China;; b National Navel Orange Engineering and Technology Research Center, Ganzhou, Jiangxi, China

**Keywords:** Encyrtidae, mitochondrial genome, *Diaphorencyrtus aligarhensis*, phylogenetic analysis

## Abstract

*Diaphorencyrtus aligarhensis* is an important natural enemy of the psyllid *Diaphorina citri* Kuwayama, a vector of the huanglongbing (HLB). Here, we sequenced and annotated the mitochondrial genome (mitogenome) of *D. aligarhensis*. This mitogenome was 16,264 bp long and encoded 13 protein-coding genes (PCGs), 22 transfer RNA genes (tRNAs), and two ribosomal RNA unit genes (rRNAs). All 13 PCGs were initiated by the ATN (ATG, ATT, ATA, and ATC) codon. All PCGs terminate with the stop codon TAA except for *cox2* and *nad1* which end with the incomplete codon T−. Phylogenetic analysis showed that *D. aligarhensis* got together with the same family species *Encyrtus infelix*, and Encyrtidae had a close relationship with Agaonidae.

*Diaphorencyrtus aligarhensis* (Shafee, Alam, and Argarwal) is an endoparasitoid of Asian citrus psyllid, *Diaphorina citri* Kuwayama (Hemiptera: Liviidae). *Diaphorencyrtus aligarhensis* was originally recorded in India and was imported into many countries as biological control agents for *D. citri* (Shafee et al. 1975; Rohrig et al. 2011).

Specimens of *D. aligarhensis* were collected from Puning City, Guangdong Province, China (23°21′N, 116°2′E, October 2018) and were stored in Entomological Museum of Gannan Normal University (Accession number GNU-DA017). After morphological identification, total genomic DNA was extracted from tissues using DNeasy DNA Extraction kit (Qiagen, Hilden, Germany). The mitogenome sequence of *D. aligarhensis* was generated using Illumina HiSeq 2500 Sequencing System (Illumina, San Diego, CA). In total, 6.9 G raw reads were obtained, quality-trimmed, and assembled using MITObim v 1.7 (Hahn et al. 2013). By comparison with the homologous sequences of other Chalcidoidea species from GenBank, the mitogenome of *D. aligarhensis* was annotated using software GENEIOUS R8 (Biomatters Ltd., Auckland, New Zealand).

The nearly complete mitogenome of *D. aligarhensis* is 16,264 bp (Genbank accession, MN274569). It contains 13 protein-coding genes (PCGs), 22 tRNA genes, 2 rRNA genes, and a partial non-coding AT-rich region. The putative control region located between *trnM* and *nad2*, which was failed to sequence and was similar to other Chalcidoidea species (Su et al. 2016; Zhu et al. 2018; Tang et al. 2019). The nucleotide composition of the mitogenome was biased toward A and T, with 81.8% of A + T content (A 46.8%, T 35.0%, C 11.5%, G 6.7%). Mitogenome of *D. aligarhensis* exhibit dramatic mitochondrial gene rearrangement which is common in Chalcidoidea species (Chen et al. 2018; Xiong et al. 2019). All 13 PCGs of *D. aligarhensis* have the conventional ATN start codons for invertebrate mitochondrial PCGs (5 ATG, 5 ATT, 2 ATA, and 1 ATC). Most of the PCGs terminate with the stop codon TAA, whereas *cox2* and *nad1* end with the incomplete codon T. The 22 tRNA genes vary from 56 bp (*trnE*) to 77 bp (*trnC*). Two rRNA genes (*rrnL* and *rrnS*) are located at *trnL1*/*trnA* and *trnA*/*trnV* regions, respectively. The lengths of *rrnL* and *rrnS* in *D. aligarhensis* are 1310 and 798 bp, with the same AT contents of 86.6%.

All 13 mitochondrial protein-coding genes sequences were extracted from the complete or nearly complete mitochondrial DNA sequences of 17 closely related taxa of Chalcidoidea, including two outgroup species from Cynipoidea and Proctotrupoidea. The phylogenetic tree was constructed using the maximum-likelihood method through raxmlGUI 1.5 (Silvestro and Michalak 2012). The newly sequenced species *D. aligarhensis* got together with the same family species *Encyrtus infelix* with high support value (BS = 100), and Encyrtidae had a close relationship with Agaonidae (Figure 1). In conclusion, the mitogenome of *D. aligarhensis* is sequenced in this study and can provide important DNA molecular data for further phylogenetic and evolutionary analysis of Chalcidoidea.

## Figures and Tables

**Figure 1.**
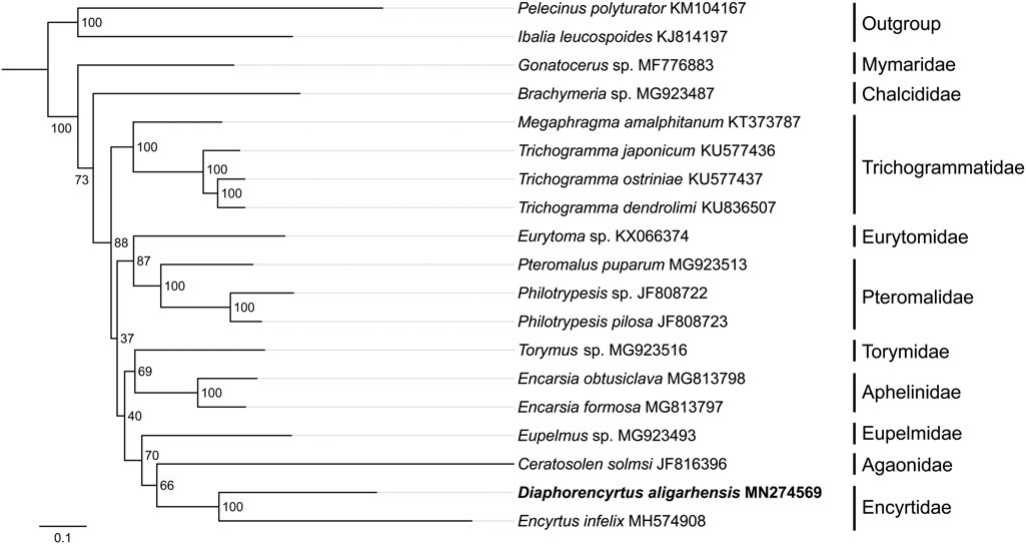
Phylogenetic relationships based on the 13 mitochondrial protein-coding genes sequences inferred from RaxML. Numbers on branches are Bootstrap support values (BS).
